# The internal structure of the infraspinatus muscle: a magnetic resonance study

**DOI:** 10.1007/s00276-022-03042-2

**Published:** 2022-11-08

**Authors:** Isabelle Jernheden, Pawel Szaro

**Affiliations:** 1 Department of Musculoskeletal Radiology, Sahlgrenska University Hospital, Göteborgsvägen 31, 431 80 Gothenburg, Sweden; 2 Department of Radiology, Institute of Clinical Sciences, Sahlgrenska Academy, University of Gothenburg, Gothenburg, Sweden; 3 Department of Descriptive and Clinical Anatomy, Medical University of Warsaw, Warsaw, Poland

**Keywords:** Infraspinatus muscle, Anatomy, Magnetic resonance imaging, Rotator cuff

## Abstract

**Purpose:**

This study aimed to describe the internal structure of the infraspinatus muscle. A secondary aim was to explore differences in internal structure between genders, sides, and correlations to demographic data.

**Methods:**

In total, 106 shoulder MRI examinations of patients between 18 and 30 years of age seeking care in 2012–2020 at The Sahlgrenska University Hospital in Gothenburg, Sweden were re-reviewed.

**Results:**

The number of intramuscular tendons centrally in the infraspinatus muscle varied between 3 and 8 (median = 5). Laterally, the number of intramuscular tendons varied between 1 and 5 (median = 2). There was no difference in the median between the genders or sides. No correlations between the number of intramuscular tendons and demographic data were found. The muscle volume varied between 63 and 249 ml with a median of 188 ml for males and 122 ml for females. There was no significant difference in volume between the sides. The muscle volume correlated with body weight (Pearson’s correlation coefficient, *r* = 0.72, *p* < 0.001) and height (*r* = 0.61, *p* < 0.001).

**Conclusion:**

The anatomical variations of the infraspinatus muscle are widespread. In the medial part of the muscle belly, the number of intramuscular tendons varied between 3 and 8, while the number of intramuscular tendons laterally varied between 1 and 5. Results of our study may help to understand the internal structure of the infraspinatus muscle and its function in shoulder stabilization.

## Introduction

Shoulder pain is common with maximal incidence rates in middle age [31] and rotator cuff pathology is common [26]. Previous research has established that rotator cuff pathology harms shoulder strength [22] and function as well as the quality of life [7].

A common feature of previous studies on rotator cuff pathology is that they target the supraspinatus rather than the other rotator cuff muscles [9, 25]. This choice of focus may be explained by supraspinatus being the far most common location for rotator cuff tears [3, 10]. However, several studies have postulated that the infraspinatus muscle may have a more prominent role in the pathogenesis of rotator cuff ruptures [14, 24] than what previous studies laid interest in mapping the internal structure of the infraspinatus muscle more precisely.

Most previous studies have been based on cadaver dissection, indicating that the structure is complex with a multipennate configuration [2, 15]. However, recent literature offers contradictory findings regarding how the muscle bundles are arranged. Kato et al. have conducted a morphologic and histologic analysis that concludes that the muscle is to be divided into two separate parts with regards to muscle fiber direction; one narrow transverse part with origin on the inferior edge of the scapular spine and one wider oblique part with origin in the infraspinous fossa [14]. In contrast to Kato et al., Bacle et al. have demonstrated that the infraspinatus muscle comprises three parts, two superficial parts and a third underlying part [1]. Other research teams have also supported such a three-part model [8]. Bacle et al. suggest that the divergent findings could be explained by yet not explored anatomical variants or dissimilar embalming techniques and clearly state that further research through imaging or in vivo is needed to confirm the three-parted muscle model [1].

This study aimed to describe the internal structure of the infraspinatus in patients between 18 and 30 years of age based on magnetic resonance imaging (MRI). A secondary exploration aimed to analyze potential differences in internal structure between genders and between the right and left sides. Moreover, the study aimed to investigate whether there is a correlation between different variants of internal structure and age, body weight, height, or body mass index (BMI).

## Materials and methods

### Data collection procedures

This descriptive study was based on shoulder MRI examinations of patients between 18 and 30 years of age seeking care in the years 2012–2020 at The Sahlgrenska University Hospital in Gothenburg, Sweden. Consequently, all included images were taken on clinical indication. The study was limited to patients between 18 and 30 years of age since young subjects seldomly have degenerative lesions [23, 29], which is desirable when aiming at a presentation of normal anatomy. Due to the descriptive design of this study, a power analysis was not applicable. Hence, the number of subjects was set to approximately 100 considering what is generally accepted in the field of anatomic studies and supposition that it would suffice to give an overview of the most common anatomical variations. A compilation of the inclusion criteria is accounted for in Table 1.

**Table 1:** Inclusion criteria for MRI examinations

Inclusion criteria	
Shoulder MRI conducted at The Sahlgrenska University Hospital	
Examination conducted in the years 2012–2020	
Patient age between 18 and 30 years at the time of the examination	

*MRI* magnetic resonance imaging

All images suggesting changes or injuries that could affect the anatomy of the infraspinatus muscle were excluded. All exclusion criteria are seen in Table 2.

**Table 2:** Exclusion criteria for MRI examinations

Exclusion criteria	Number of cases
Injuries of the infraspinatus muscle or adjacent structures	8
Tumors in the infraspinatus muscle or adjacent structures	4
Surgical interventions disrupting the normal anatomy	5
Developmental disorders of the rotator cuff	1
History of radiation therapy in the examined area	0
Missing data regarding body weight and/or height	34
Intraarticular contrast in the glenohumeral joint	23
Suboptimal image quality	6
Examinations with insufficient coverage of the infraspinatus muscle	14
More than one examination of the same shoulder	2
All exclusion criteria summarized	97

*MRI* magnetic resonance imaging

Examinations were reviewed in the imaging platform used at the hospital, AGFA^©^ PACS (picture archiving and communication system), where images, referrals, and radiology reports were available. Together with the radiology report, a routine questionnaire stating the patient's body weight and height was archived. The examinations were collected based on examination date, starting at the last conducted examination of 2020, working backward in time until the chosen number of subjects was reached.

The patient's gender and age at the examination time were decided by personal identity number. Body weight and height were read out from the routine questionnaire archived together with the radiology report. All data were noted in a Microsoft Excel^©^ spreadsheet. Microsoft Excel^©^ was programmed to calculate BMI from body weight and height.

The radiological data were obtained through reviewing the examinations. First, whether the examination concerned the right or left shoulder was noted. Then length measurements were done using the tool Measuring and angle measurements were done using the tool Angle. All measurements were conducted by a beforehand established protocol to keep measuring points as homogenous as possible. The protocol of measurements is described in detail in the next paragraph.

### Protocol of measurements

**Fig. 1:**
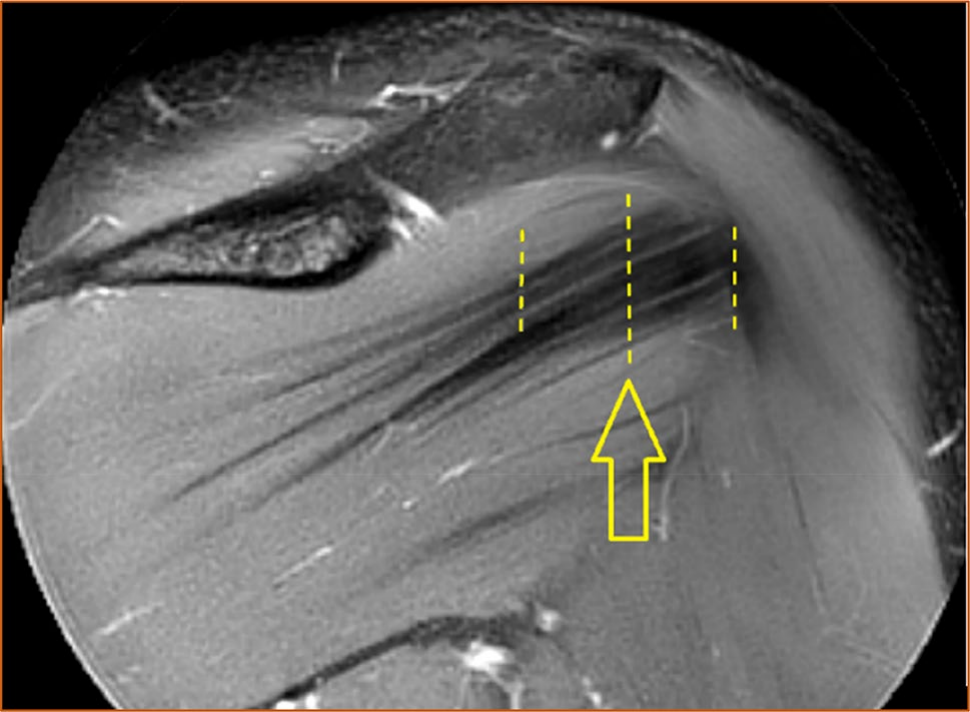
The intramuscular tendons of the infraspinatus muscle were counted in a vertical line (dashed) under the deltoid tubercle of the scapular spine (arrow) in the coronal view. This line was labeled "the central measuring point". T2-weighted image, spectral adiabatic inversion recovery. Coronal section

**Fig. 2:**
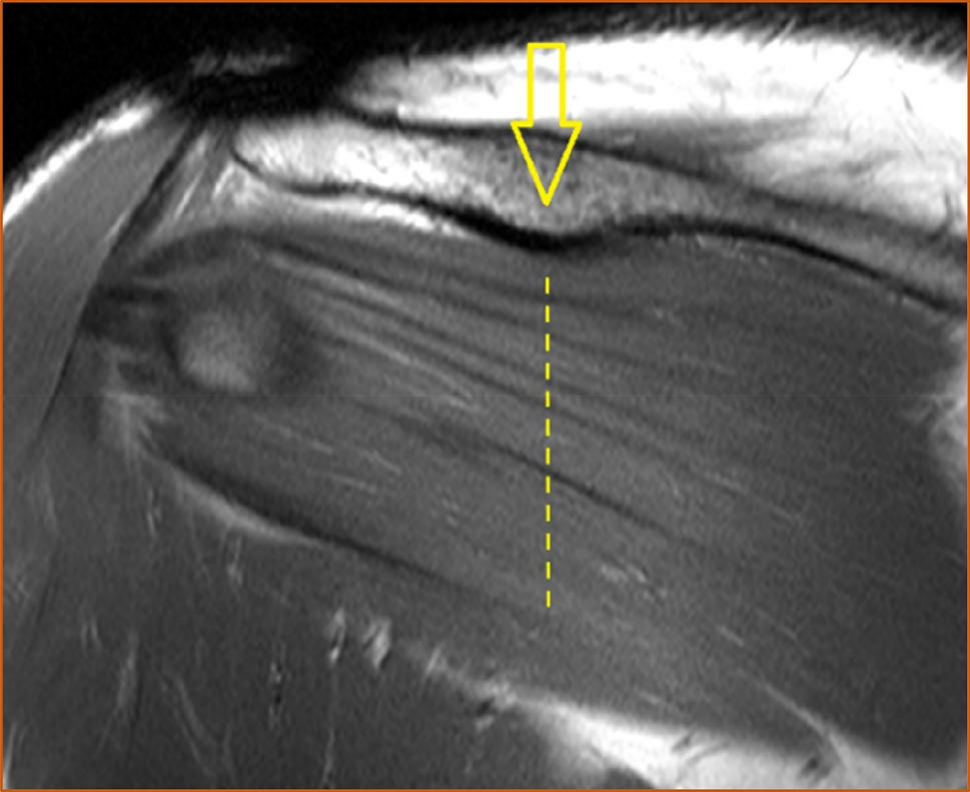
The intramuscular tendons of the infraspinatus muscle were counted in a vertical line (dashed above the arrow) midway between the glenohumeral joint space (left-most line) and the insertion of the muscle (right-most line) in the coronal view. The line above the arrow was labeled "the lateral measuring point". T1-weighted image, turbo spin echo. Coronal section

The number of distinguishable intramuscular tendons was counted in the coronal view at two sites, both in a vertical line under the deltoid tubercle of the scapular spine (Fig. 1) and in a vertical line midway between the glenohumeral joint space and the insertion of the infraspinatus muscle (Fig. 2). Henceforth in this report, the vertical line under the deltoid tubercle will be labeled the central measuring point and the vertical line midway between the glenohumeral joint space and the insertion of the infraspinatus muscle will be labeled the lateral measuring point. The intramuscular tendon was defined as a band of dense fibrous connective tissue that could be distinguished from the surrounding muscle fibers on MRI. Each separate band was labeled an intramuscular tendon, irrespective of whether the band inserted directly into the bone or converged to other bands of connective tissue before reaching the bone. Only intramuscular tendons deriving from the infraspinatus muscle were counted, not the adjacent intramuscular tendons of the teres minor muscle. The most occurring combinations of the number of intramuscular tendons at the two measuring points were presented as anatomical variants.

**Fig. 3:**
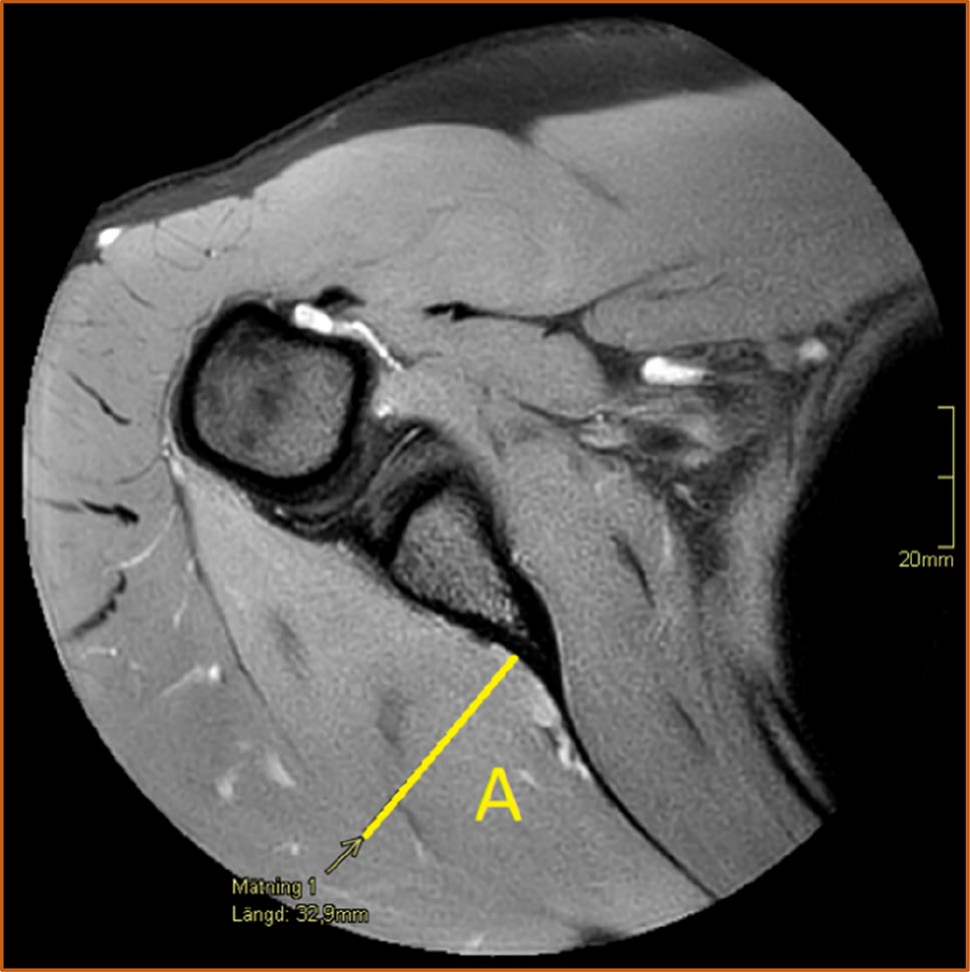
The anteroposterior diameter (A) of the infraspinatus muscle measured in the axial view. Proton density-weighted image, spectral adiabatic inversion recovery. Axial section

**Fig. 4:**
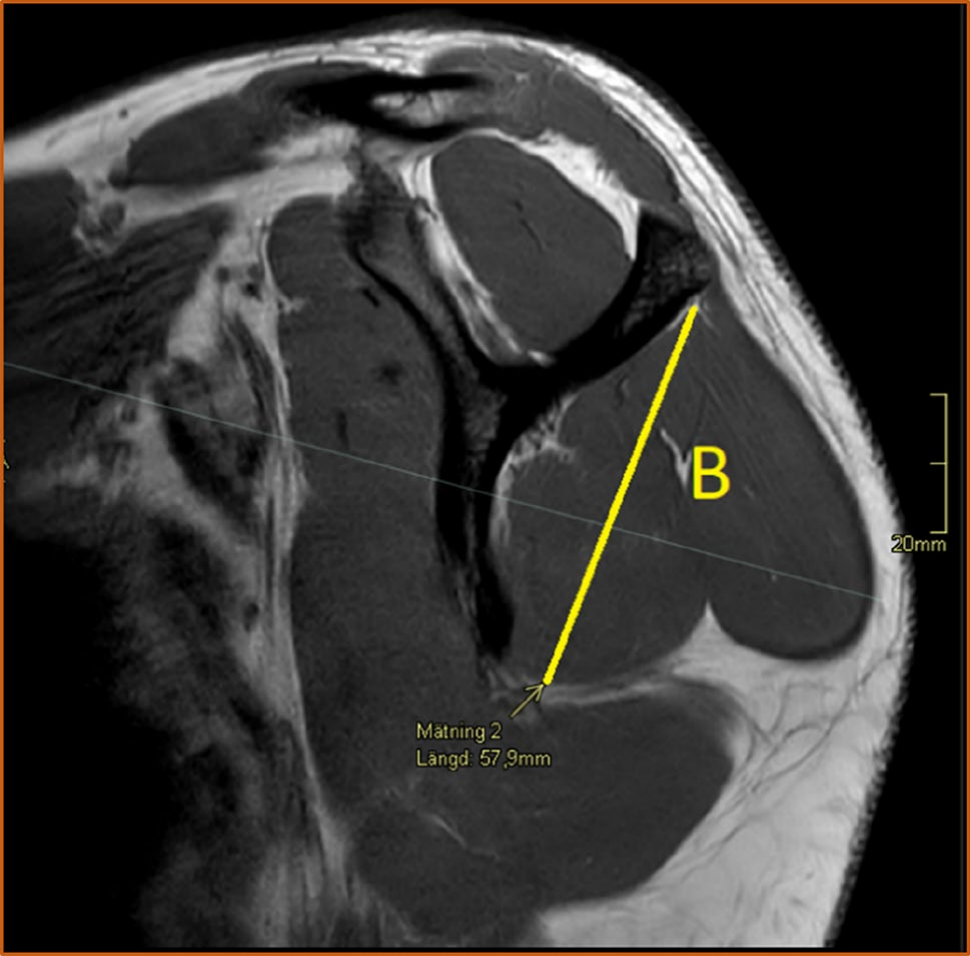
The craniocaudal diameter (B) of the infraspinatus muscle measured in the sagittal view. T2-weighted image, turbo spin echo. Sagittal section

**Fig. 5:**
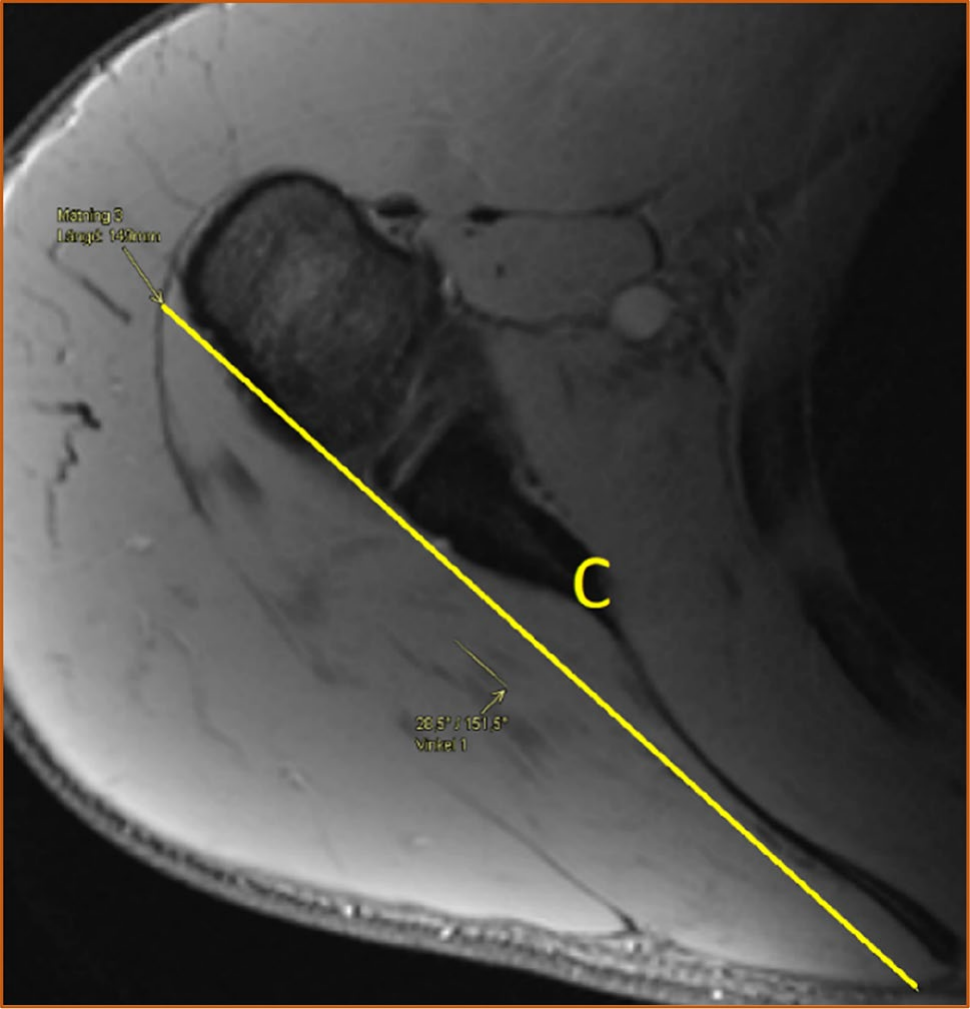
The lateromedial diameter (C) of the infraspinatus measured in the axial view. Proton density-weighted image, spectral adiabatic inversion recovery. Axial section

The shape of the infraspinatus muscle was approximated to a tri-axial spheroid, making it possible to calculate the muscle volume (*V*) from three diameters (A, B, and C) using the formula *V* = *π*/6*ABC. The anteroposterior diameter (A) was estimated by measuring the widest portion of the muscle belly seen in the axial view (Fig. 3). The craniocaudal diameter (B) was measured in the sagittal view at approximately the same location as the anteroposterior diameter and perpendicular to the axial section (Fig. 4). The lateromedial diameter (C) was estimated as the distance between the origin of the muscle in the scapula and the lateral aspect of the muscle belly seen in the axial view (Fig. 5).

**Fig. 6:**
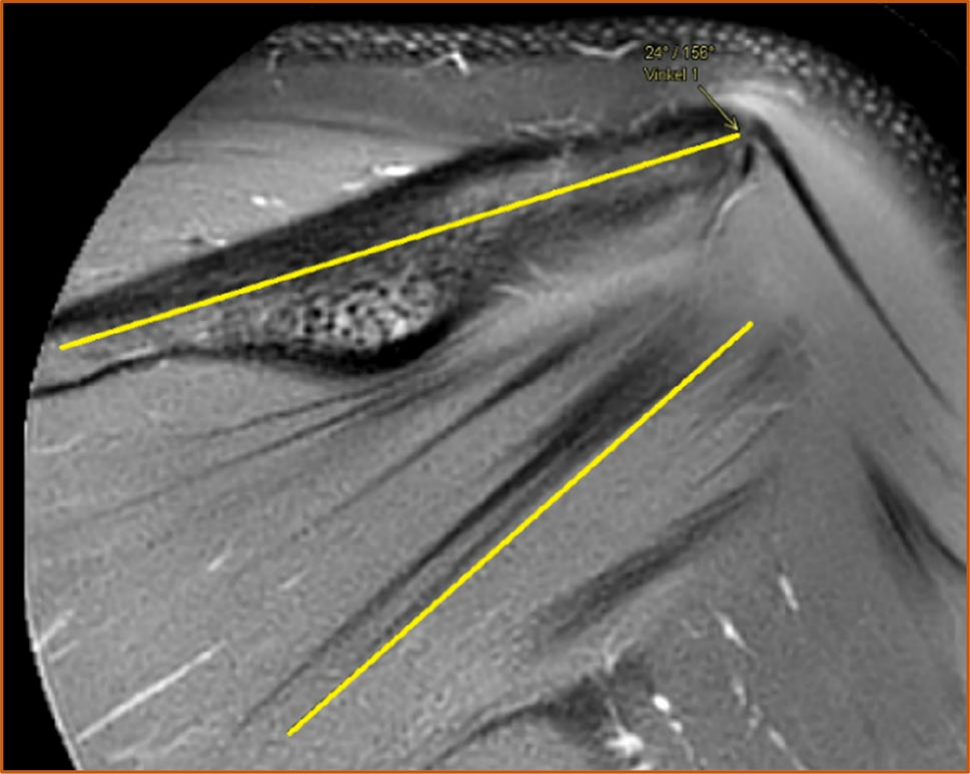
The angle between the longitudinal axis of the scapular spine and the lower portion of the most inferior tendon of the infraspinatus muscle seen in the coronal view. T2-weighted image, spectral adiabatic inversion recovery. Coronal section

**Fig. 7:**
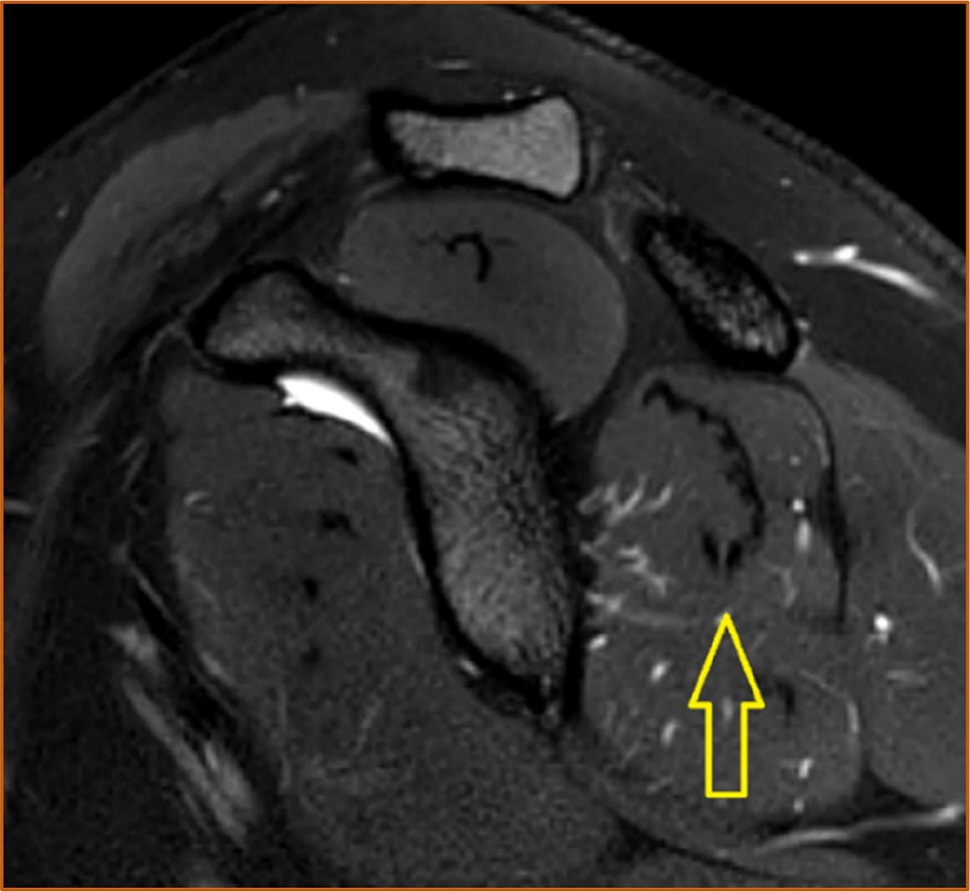
The position of the intramuscular tendons of the infraspinatus muscle seen in the sagittal view. In this case, they were considered entirely intramuscular. T2-weighted image, spectral adiabatic inversion recovery. Sagittal section

The angle between the longitudinal axis of the scapular spine and the lower portion of the most inferior intramuscular tendon of the infraspinatus muscle was also measured (Fig. 6). The thought behind measuring this angle was to indicate the direction of the tendons' course. The scapular spine was selected as a reference point since it is clearly visible in the coronal view and does not significantly change position in relation to the muscle throughout the movement in the arm. Lastly, the position of the intramuscular tendons of the infraspinatus muscle was judged from examination in the sagittal view as either entirely intramuscular or partially superficial (Fig. 7). All images taken in sagittal view were examined. If any portion of the intramuscular tendons had a superficial course along the posterior surface of the muscle, they were considered partially superficial. The tendons were considered entirely intramuscular if no superficial portion was seen in the sagittal view.

Two observers examined all images. In cases of diverging observations of the number of intramuscular tendons or their position, the discussion was held to reach a consensus. For continuous scale data, i.e., the measurement of diameters and the angle, a mean value of the two observers' data was calculated and used in the statistical analysis.

### Statistical methods

All statistical analyses were done in the software program SPSS^©^ (Statistical Package for the Social Sciences) version 28.0. To establish whether the continuous variables were normally distributed, histograms were constructed, and Shapiro–Wilk test was conducted. The variance was analyzed with Levene's Test for equality of variances. Student's *t *test was used to analyze differences in mean values for normally distributed data. Continuous variables deviating from normal distribution as well as discrete variables were instead analyzed with Mann–Whitney *U*-test. Significant results were further analyzed with independent samples median test and quantile regression. Whether there was any association between gender or side and the prevalence of a superficial portion of the intramuscular tendons was analyzed with Fisher's exact test and the Chi-squared test. The threshold for statistical significance was throughout all tests set to *p* < 0.05.

The correlation between demographic data and the measured variables was investigated with Pearson's correlation coefficient (*r*) and Spearman's rank correlation coefficient (*r*_s_). Pearson's correlation coefficient was chosen for continuous data following a normal distribution. Spearman's rank correlation coefficient was chosen for continuous data diverging from a normal distribution and for discrete numerical data. Both correlation coefficients were interpreted as follows: |*r*|< 0.2 = very weak relationship, 0.2 ≤|*r*|< 0.4 = weak relationship, 0.4 ≤|*r*|< 0.6 = moderate relationship, 0.6 ≤|*r*|< 0.8 = strong relationship, and |*r*|≥ 0.8 = very strong relationship [4].

As earlier described, all images were independently examined by two observers, and in cases of different observations, the discussion was held to reach a consensus for discrete and binary data, and for continuous data, a mean value was calculated. Nonetheless, it is of interest to assess the inter-rater variability to verify the reproducibility of the study. This was done by calculating Cohen's kappa (*κ*) for binary variables and Cohen's weighted kappa (*κ*_w_) for discrete numerical variables. The quadratic weighting system was used. Interpretation of the kappa values was done with the intervals of agreement established by Landis and Koch: poor (*κ* < 0), slight (*κ* = 0–0.20), fair (*κ* = 0.21–0.40), moderate (*κ* = 0.41–0.60), substantial (*κ* = 0.61–0.80), and almost perfect agreement (*κ* = 0.81–1.00) [19]. The intraclass correlation coefficient (ICC) of the type two-way random-effects model was applied for continuous numerical measurements. This type of ICC makes possible generalization of the results to other raters with a similar degree of education and experience. The assessment basis was set to the mean of the raters, and the definition of the agreement was absolute numerical agreement. The interpretation was made according to the guidelines of Koo and Li: poor (ICC < 0.50), moderate (0.50 ≥ ICC < 0.75), good (0.75 ≥ ICC < 0.90), and excellent reliability (ICC ≥ 0.90) [18].

### MRI sequences

The MRI sequences vary among examinations since they were conducted during 9 years and by different machines. However, all images were taken with a machine from either Siemens^©^ or Philips^©^ with a 1.5 or 3 T magnetic field together with a specific shoulder coil that enhances image quality. Table 3 shows the sequences that were reviewed in this study.

**Table 3:** MRI sequences in this study

Sequence	TE (ms)	TR (ms)	FOV	Voxel size (Ax/Sag/Cor)
PD TSE SPAIR Ax	30	2700–5000	140 × 140 × 104 mm	0.50 × 0.50 × 3.00 mm
T2 TSE SPAIR Sag	70	3000–5000	140 × 140 × 84 mm	0.50 × 0.50 × 3.00 mm
T1 TSE Cor	9	450–750	160 × 140 × 69 mm	0.45 × 0.58 × 3.00 mm
T2 TSE SPAIR Cor	45	3000–5000	140 × 140 × 87 mm	0.52 × 0.52 × 3.00 mm

*Ax* axial section, *Cor* coronal section, *FOV* field of view, *MRI* magnetic resonance imaging, *PD* proton density, *Sag* sagittal section, *SPAIR* spectral adiabatic inversion recovery, *TE* echo time, *TR* repetition time, *TSE* turbo spin echo

The position of the shoulder and arm was secured by elastic cushions which were placed between the coil reflecting the shape of the arm with the patient.

### Nomenclature and terminology

In the field of morphological studies, there is a general lack of consistency in the nomenclature of tendons and their substructures [11]. In this study, we used the term infraspinatus tendon for the terminal tendon inserted on the tuberculum majus, while in the muscle belly, intramuscular tendinous were identified.

### Ethics

The Swedish Ethical Review Authority has approved the study and waived the need for informed consent (Dnr: 2020-05954).

## Results

### Demographics of the study population

After applying of exclusion and inclusion criterion, we included 106 examinations in this study. To reach this number, examinations back until the date 2012-11-16 were reviewed. Table 4 presents the distribution of gender and examined side.

**Table 4:** The distribution of gender and examined side in the study population

	Male	Female	*Σ* (%)
Right	39	14	53 (50)
Left	34	19	53 (50)
*Σ* (%)	73 (68.9)	33 (31.1)	106 (100)

*Σ* summation

The demographic characteristics of the included subjects are compiled in Table 5. The mean age of the subjects was 23.6 years. The mean BMI was 24.2.

**Table 5:** Demographic data of the study population

	Age (years)	Weight (kg)	Height (cm)	BMI (kg/m^2^)
Mean	23.6	77.2	178	24.2
Minimum	18	49	155	16
Maximum	29	113	196	35.9
Median	23.5	76.5	180	24.1
SD	3.3	14.4	8.6	3.4
SD error of mean	0.3	1.4	0.8	0.3

*BMI* body mass index, *SD* standard deviation, *SD error of mean* standard error of the mean

### Excluded MRI examinations

**Fig. 8:**
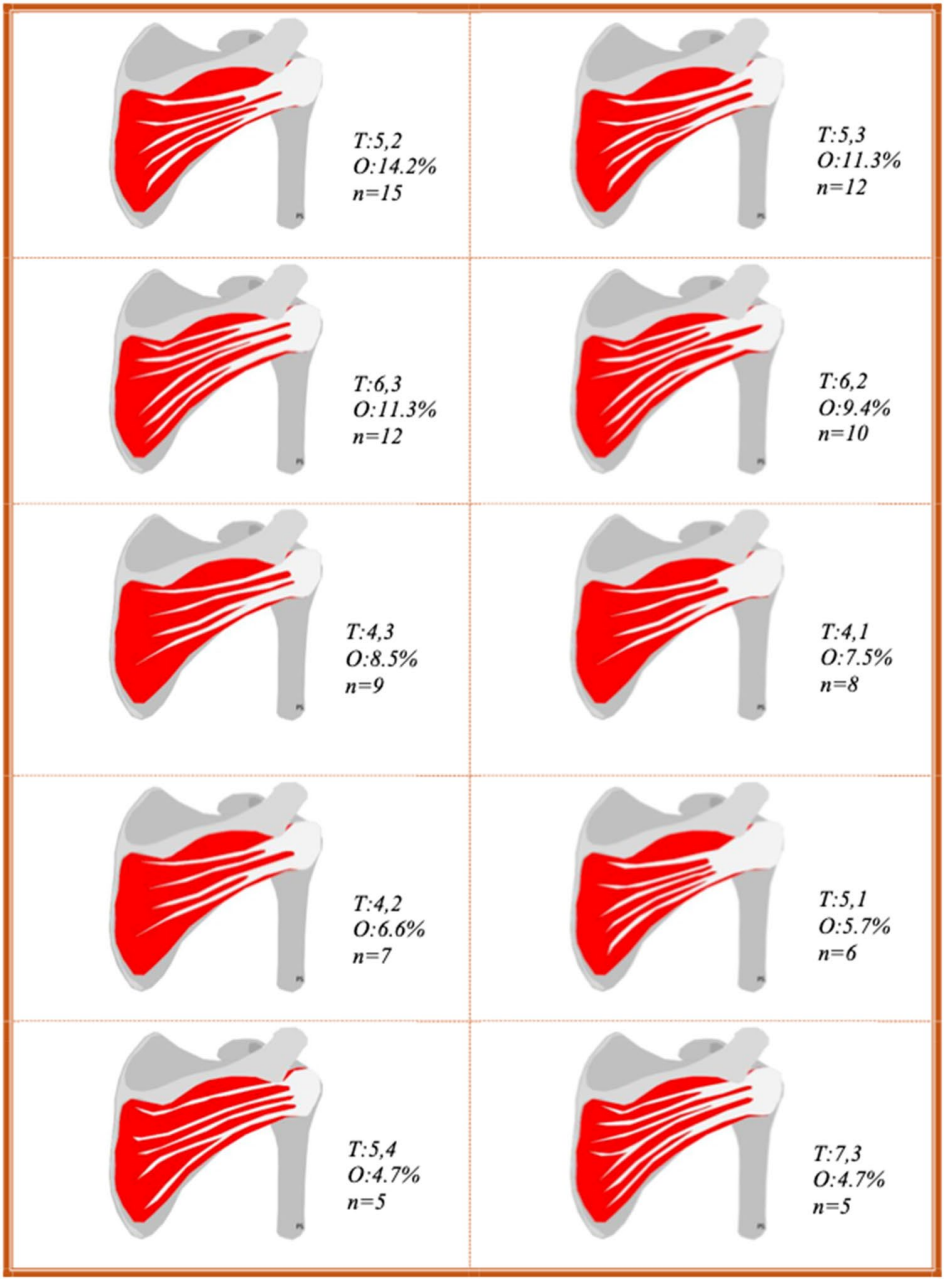
Illustrations of the ten most frequently occurring anatomical variants of intramuscular tendons in this study. For each variant, the number of intramuscular tendons are denoted as T: number of intramuscular tendons at the central measuring point, number of intramuscular tendons at the lateral measuring point. O, occurrence; n, number of examinations. The illustrations were drawn by PS

In total, 97 examinations were excluded from the study. The distribution of excluded cases is illustrated in Table 2. A relevant annotation is that except for these 97 examinations, additionally 44 examinations were excluded from the calculation of muscle volume. The reason for this was the absence of either axial or sagittal projections, which forbid measurement of the lateromedial or craniocaudal diameter, respectively. However, the analysis of the number of intramuscular tendons and their position and the measurement of the angle were done on all 106 included examinations (Fig. 8).

Tables 6 and 7 present the prevalence of each number of intramuscular tendons at the two measuring points as separate observations.

**Table 6:** The prevalence of each number of intramuscular tendons at the central measuring point

Intramuscular tendons at the central measuring point	3	4	5	6	7	8
%	3.8	22.6	36.8	25.5	9.4	1.9
*N*	4	24	39	27	10	2

% occurrence, *n* number of examinations

**Table 7:** The prevalence of each number of intramuscular tendons at the lateral measuring point

Intramuscular tendons at the lateral measuring point	1	2	3	4	5
%	17.9	35.8	36.8	8.5	0.9
*N*	19	38	39	9	1

% occurrence, *n* number of examinations

**Fig. 9:**
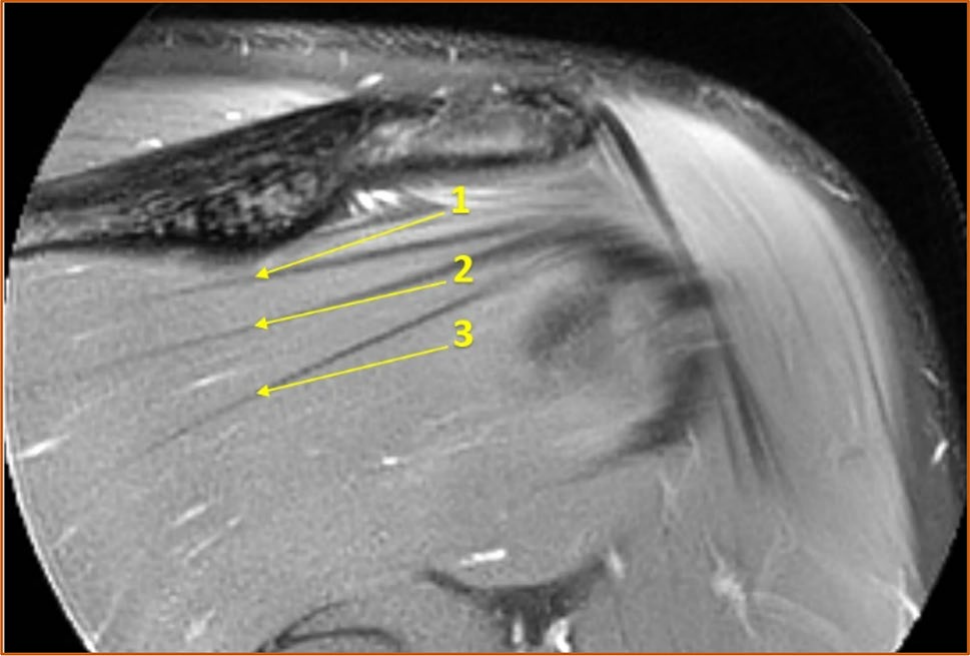
Example of a muscle with many intramuscular tendons at the central measuring point. T2-weighted image, spectral adiabatic inversion recovery. Coronal section

**Fig. 10:**
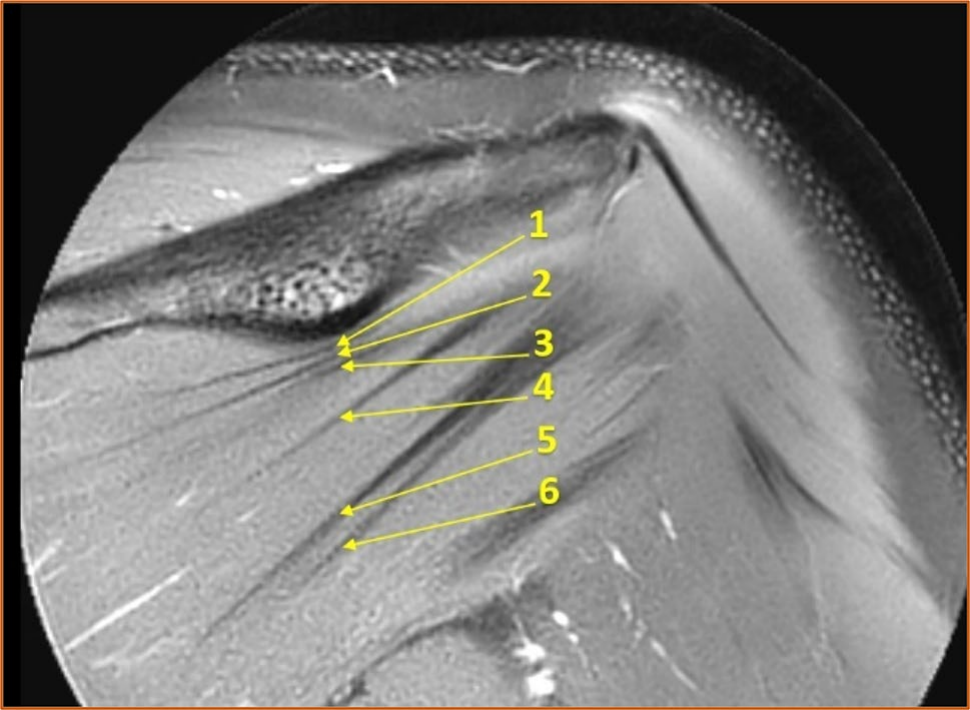
Example of a muscle with few intramuscular tendons at the central measuring point. T2-weighted image, spectral adiabatic inversion recovery. Coronal section

The median number of intramuscular tendons at the central measuring point was five, ranging from three to eight among males (standard deviation, SD = 1.0) and from three to seven among females (SD = 1.1). A difference in distribution between the genders was seen (*p* = 0.019), but there was no difference in median (*p* = 1.00). When examining the distribution in greater detail, it was found that males had one tendon more than females at both the lower quartile (95% CI 0.45–1.55), *p* < 0.001 and the upper quartile (95% CI 0.45–1.55), *p* < 0.001. No difference in distribution was observed between the right (SD = 1.1) and left (SD = 1.0) sides (*p* = 0.50). Figure 9 illustrates an example of a muscle with many intramuscular tendons at the central measuring point and Fig. 10 illustrates an example of a muscle with few intramuscular tendons at the central measuring point.

At the lateral measuring point, the median number of intramuscular tendons were two for both genders, varying between one and four for males (SD = 0.8) and between one and five for females (SD = 1.1). No difference in distribution between the genders was detected (*p* = 0.82). Neither a difference in distribution between the right (SD = 1.0) and left (SD = 0.8) sides was observed (*p* = 0.49).

No correlation between age, body weight, height, or BMI and the number of intramuscular tendons was found, neither at the central or lateral measuring point.

### Muscle volume

**Fig. 11:**
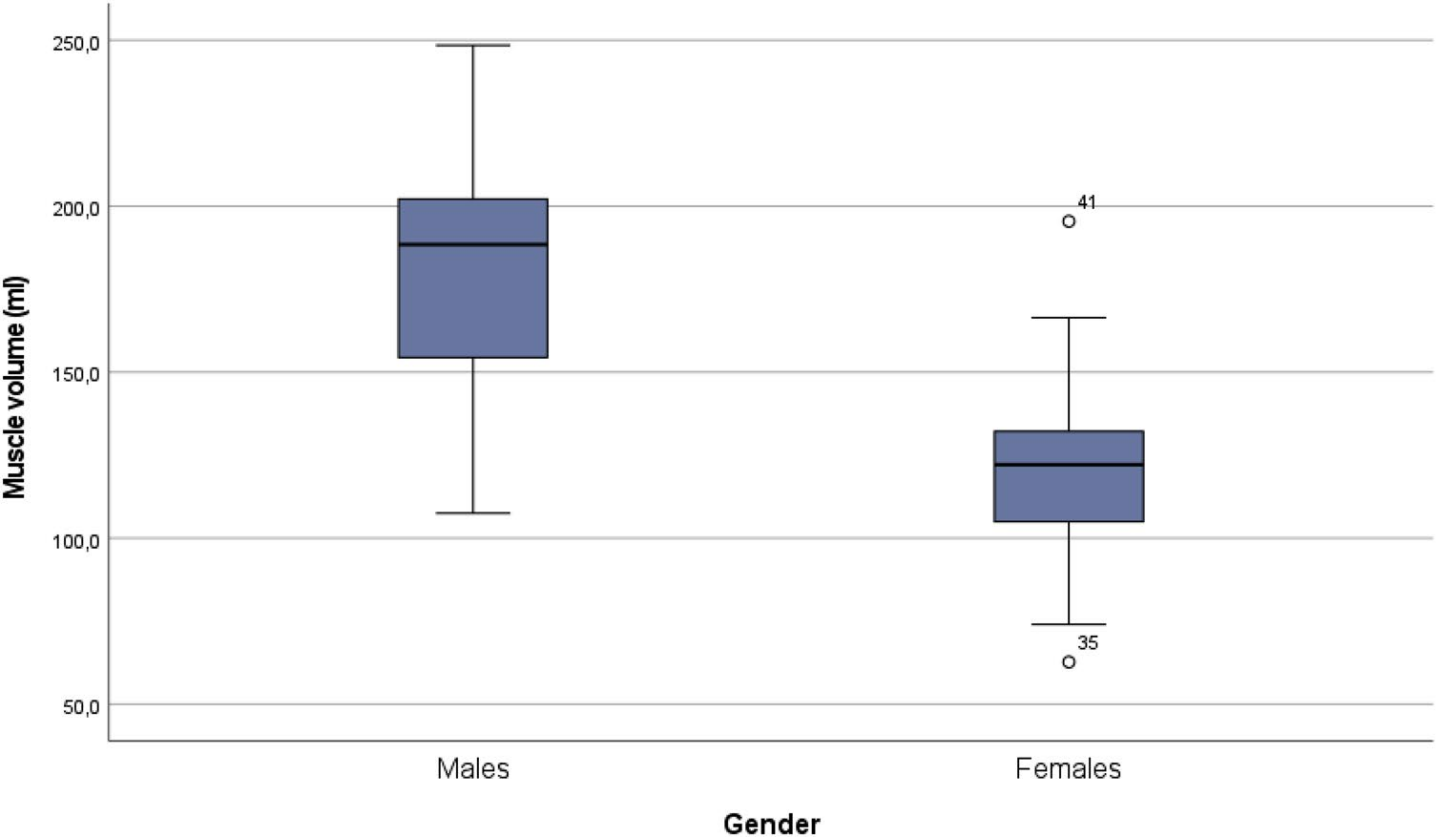
Boxplot illustrating the muscle volume distribution for males and females in the reviewed examinations. The difference in median volume between the genders was 68 ml (95% CI 44.1–91.0), *p* < 0.001

The median muscle volume was 188 ml for males (SD = 33; the number of examinations, *n* = 42) and 122 ml for females (SD = 32, *n* = 20), see Fig. 11. The difference in median muscle volume between the genders was 68 ml (95% CI 44.1–91.0), *p* < 0.001. No statistically significant difference in mean volume for the right (*n* = 31) and left (*n* = 31) side was found, − 0.07 ml (95% CI − 22.2 to 22.2), *p* = 1.00.

**Fig. 12:**
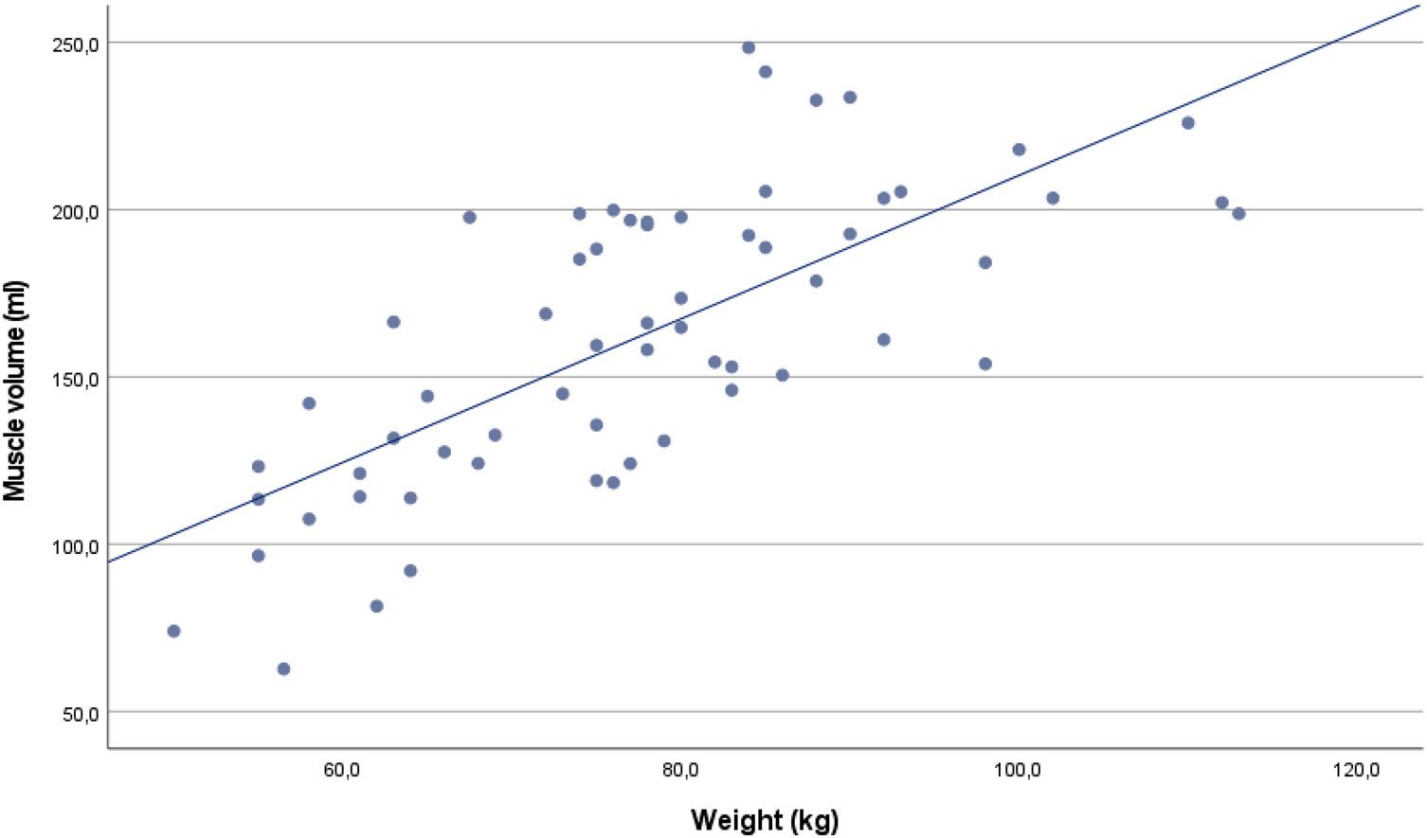
Scatter plot illustrating the correlation between body weight (kg) and muscle volume (ml). Pearson's correlation coefficient, *r* = 0.72

The section below accounts for the study of correlations between muscle volume and the numerical demographic data. The result of the correlation analysis of the normally distributed variables is summarized in Table 8. Both correlation coefficients were over 0.6 and hence interpreted as strong. The strongest relationship was observed between body weight and muscle volume (*r* = 0.72) and is illustrated in Fig. 12.

**Table 8:** Pearson's correlation coefficient for muscle volume and demographic data

Variable	Pearson's correlation coefficient (*r*)	95% CI lower bound	95% CI upper bound	*P* value
Weight	0.72	0.57	0.82	< 0.001
Height	0.61	0.42	0.74	< 0.001

*CI* confidence interval

Correlations between muscle volume and the non-normally distributed demographic data are shown in Table 9. There was a strong significant relationship between BMI and muscle volume, *r*_s_ = 0.65. The correlation between age and muscle volume was not statistically significant.

**Table 9:** Spearman's rank correlation coefficient for muscle volume and demographic data

Variable	Spearman's rank correlation coefficient (*r*_s_)	95% CI lower bound	95% CI upper bound	*P* value
Age	0.25	− 0.01	0.47	0.05
BMI	0.65	0.48	0.78	< 0.001

*CI* confidence interval

Turning now to the correlation analysis between muscle volume and the other measured variables, a relationship between muscle volume and the number of intramuscular tendons at the central measuring point was found. However, the correlation was weak. There were no statistically significant correlations between muscle volume and the number of intramuscular tendons at the lateral measuring point or between muscle volume and the measured angle. For exact values, see Tables 10 and 11.

**Table 10:** Spearman's rank correlation coefficient for muscle volume and the number of intramuscular tendons at the two measuring points

Variable	Spearman's rank correlation coefficient (*r*_s_)	95% CI lower bound	95% CI upper bound	*P* value
Intramuscular tendons at the central measuring point	0.36	0.11	0.56	0.01
Intramuscular tendons at the lateral measuring point	0.16	− 0.10	0.40	0.21

*CI* confidence interval

**Table 11:** Spearman's rank correlation coefficient for muscle volume and the measured angle

Variable	Pearson's correlation coefficient, r	95% CI lower bound	95% CI upper bound	*P* value
The angle between the most inferior tendon of the muscle and the scapular spine	− 0.16	− 0.39	0.10	0.22

*CI* confidence interval

### The angle between the most inferior tendon and the scapular spine

**Fig. 13:**
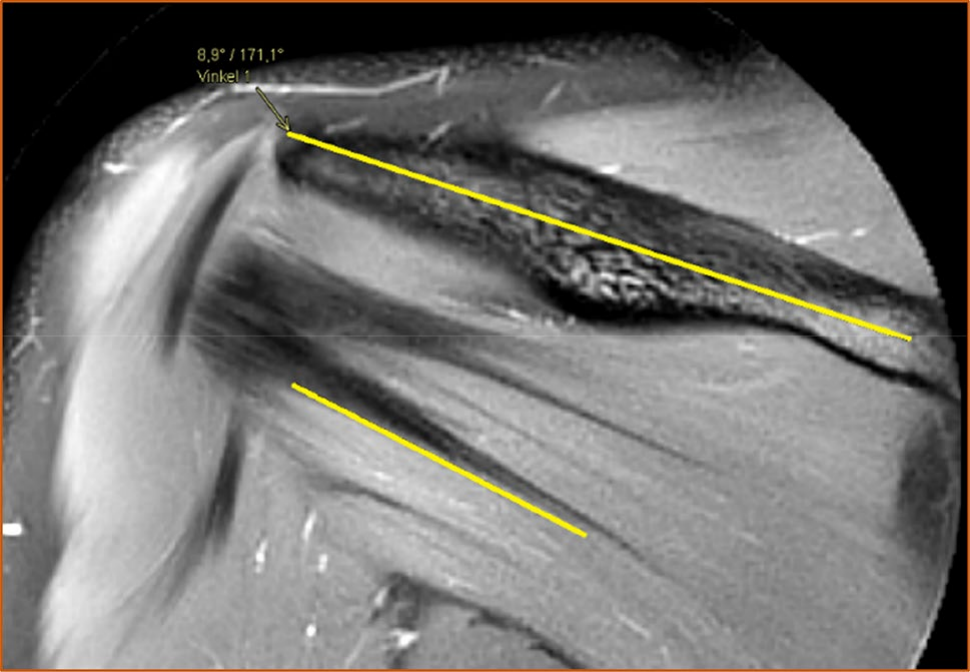
Example of an examination with a narrow angle between the most inferior tendon of the infraspinatus muscle and the scapular spine. T2-weighted image, spectral adiabatic inversion recovery. Coronal section

**Fig. 14:**
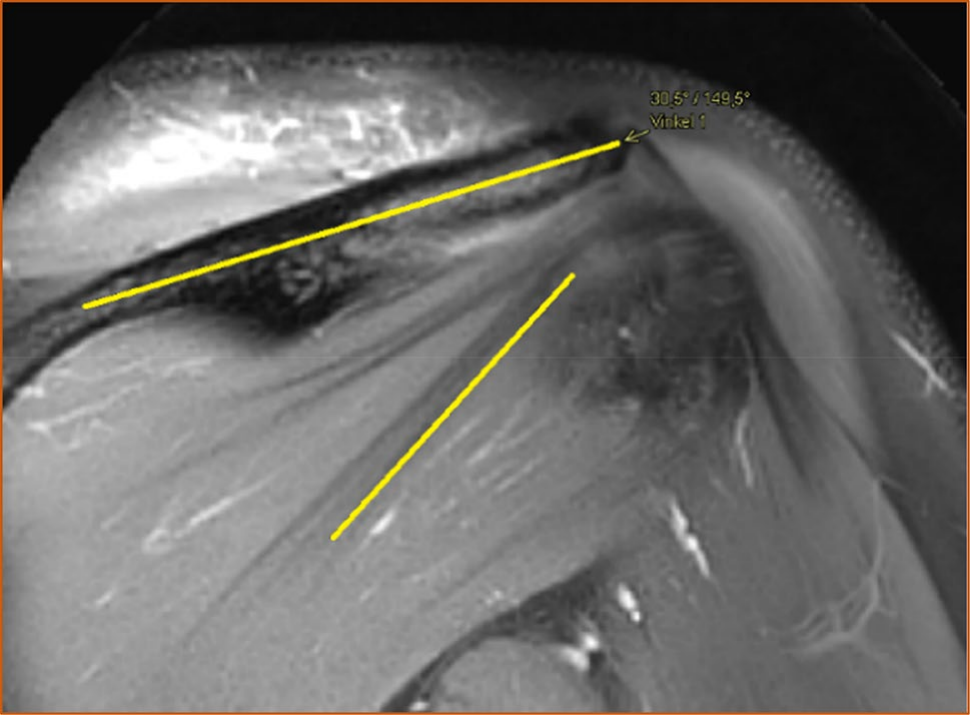
Example of an examination with a wide angle between the most inferior tendon of the infraspinatus muscle and the scapular spine. T2-weighted image, spectral adiabatic inversion recovery. Coronal section

The mean angle between the most inferior tendon and the scapular spine was 22.0°. There was no significant difference in means between the genders, − 0.51° (95% CI − 3.5 to 2.5), *p* = 0.74. The minimal value was 5.8° and the maximal value was 39.2°. An example of a narrow angle is shown in Fig. 13 and an example of a wide angle is shown in Fig. 14.

There was no significant difference in mean angle between the sides, − 1.6° (95% CI − 4.4 to 1.2), *p* = 0.27. No significant correlation was found between age, body weight, height or BMI and the measured angle.

### Localization of the intramuscular tendons

**Fig. 15:**
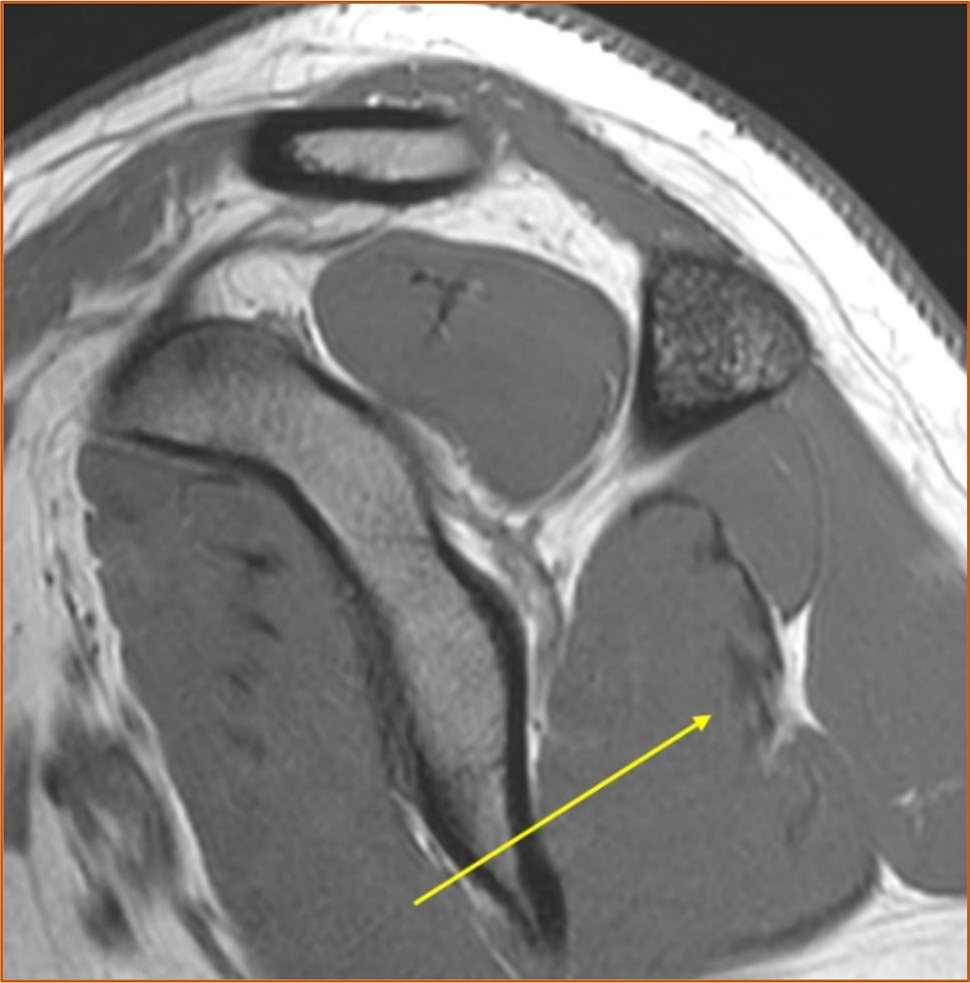
Example of intramuscular tendons that were judged as partly superficial, T1-weighted image. Sagittal section

**Fig. 16:**
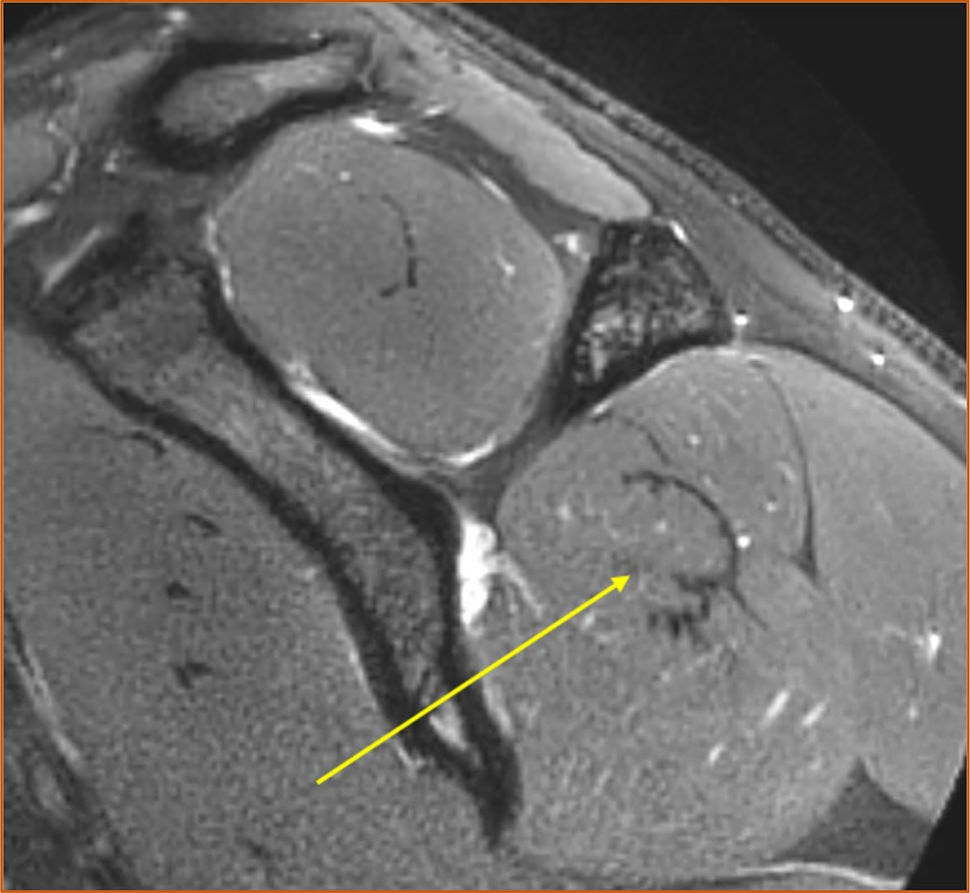
Example of intramuscular tendons that were judged as entirely intramuscular. T2-weighted image, spectral adiabatic inversion recovery. Sagittal section

In 95 of 106 examinations (89.6%), the intramuscular tendons were considered entirely intramuscular. Partly superficial intramuscular tendons were observed in 6 of 73 men (8.2%) and in 5 of 33 women (15.2%). Fischer's exact test did not prove any statistically significant association between gender and the presence of superficial intramuscular tendons (*p* = 0.31). The superficial intramuscular tendons were seen in 4 of 53 right shoulders (7.5%) and in 7 of 53 left shoulders (13.2%), but the Chi-squared test showed no association between superficial tendon position and side (*p* = 0.34). An example of partly superficial intramuscular tendons is viewed in Fig. 15 and an example of entirely intramuscular tendons is viewed in Fig. 16.

### Agreement between observers

The ICC was over 0.90 for all analyzed variables by a good margin, which corresponds to excellent reliability. The exact values of the ICC are presented in Table 12. All results were statistically significant with *p* < 0.001.

**Table 12:** The intraclass correlation coefficient for continuous variables

Variable	Intraclass correlation coefficient (ICC)	95% CI lower bound	95% CI upper bound
Anteroposterior diameter (A)	0.99	0.98	0.99
Craniocaudal diameter (B)	0.93	0.73	0.97
Lateromedial diameter^©^	0.98	0.96	0.99
The angle between the most inferior tendon and the scapular spine	0.99	0.97	0.99

All results were statistically significant with *p* < 0.001. ICC ≥ 0.90 was interpreted as excellent reliability

*CI* confidence interval

The *κ*_w_ reached almost perfect agreement (*κ* = 0.81–1.00) for both analyzed variables, *p* < 0.001. The exact values of *κ*_w_ are seen in Table 13.

**Table 13:** Cohen's weighted kappa for discrete numerical variables

Variable	Cohen's weighted kappa (*κ*_w_)	95% CI lower bound	95% CI upper bound
Intramuscular tendons at the central measuring point	0.97	0.95	0.99
Intramuscular tendons at the lateral measuring point	0.99	0.97	1.00

*κ*_w_ 0.81–1.00 was interpreted as almost perfect agreement. Both results were statistically significant with *p* < 0.001

*CI* confidence interval

The evaluation of the position of the intramuscular tendons as either entirely intramuscular or partly superficial was identical for the two observers throughout all 106 examinations. Hence, *κ* was 1.0 (95% CI 1.0–1.0), *p* < 0.001, which corresponds to almost perfect agreement.

## Discussion

This study was the first to describe the internal structure and anatomical variants of the infraspinatus muscle based on a large group of subjects and an in vivo method. The most important finding of this study was that the number of intramuscular tendons varied widely among subjects, especially at the central measuring point. Likewise, the muscle volume and the measured angle had a great spread. We used MRI as an in vivo method, which is used in clinical practice allowing the differentiation between tissues and thus counting the number of intramuscular tendons as done in this study. Another strength of MRI is that it has already been evaluated as reliable as an examination method of the rotator cuff muscles, both regarding intra-rater reliability and inter-rater reliability [13, 30]. Our study reflected this in an ICC of excellent reliability and a Cohen's kappa of almost perfect agreement throughout all examined variables.

### Number of intramuscular tendons

The number of intramuscular tendons at the central and lateral measuring points was presented as different anatomical variants. We revealed many anatomical variants; the most frequently was the one with five intramuscular tendons centrally and two intramuscular tendons laterally, but it only made up a little less than 15% of examined shoulders. These findings underline that the description of the infraspinatus as a flat muscle belly converging into one single broad tendon, as commonly seen in anatomical atlases, is a major simplification.

As described in the introduction, muscle architecture is decisive for function [14, 21]. The number of intramuscular tendons is one dimension of muscle architecture. The tendons have myotendinous junctions where the muscle fibers are attached [16]. Hence, it is plausible to assume that muscles with different numbers of tendons also have different compositions of myotendinous junctions. The myotendinous junctions are interesting since they enable force transmission from muscle fibers to tendons [16]. From this reasoning, it could be suggested that muscles with different numbers of intramuscular tendons should have slightly diverse physiological functions. One could speculate that such a variation in function might indicate that the physiotherapy recommended for patients with rotator cuff tendinopathy should be individually adapted to the internal structure of the infraspinatus muscle. Another speculation is that the variance in structure may cause some individuals to be more prone to develop rotator cuff tendinopathy than others. This association would be reasonable since the myotendinous junctions substantially increase the contact area between muscle fibers and intramuscular tendons, which is important for regulating tension and reducing stress in the musculotendinous system [16, 17].

Our objective behind studying the intramuscular tendons on MRI was threefold. One aspect was that we wanted to examine the subject in vivo, and the second, was to examine younger subjects than what is permitted by cadaver studies. The third intent was to enable the examination of a larger number of subjects. These conditions were necessary for the mapping of possible anatomical variants. However, the choice of MRI as a method also confined us to measuring other variables than those commonly examined in musculotendinous studies [1, 14]. To the best of our knowledge, no earlier study has focused on the number of intramuscular tendons rather than the muscle fiber direction and the length of the intramuscular tendons. This makes it difficult to compare our results with previous research findings. Nonetheless, the presence of different variants of internal structure, which previous studies have suggested, was supported by our results [1].

There was no evidence of the correlation between demographic data and the number of intramuscular tendons. The significant result of the Mann–Whitney *U*-test for genders and the number of intramuscular tendons at the central measuring point is not to be interpreted as a significant difference in the median but as a slight difference in distribution. Considering that males had a larger median muscle volume than females, at the same time as a correlation between muscle volume and the number of intramuscular tendons was found, the difference in distribution between genders is reasonable. Still, it should be emphasized that the correlation between the number of intramuscular tendons at the central measuring point and the muscle volume was weak. Therefore, it should be interpreted with caution.

### Muscle volume

Regarding the muscle volume, the main finding was that there was large individual variability in the volume of the infraspinatus. Also, males had a significantly larger median muscle volume (188 ml) than females (122 ml). Comparing these results with the findings of the previous studies is delicate since muscle volume can be measured by various techniques and under different assumptions. Most previous studies have been based on the dissection of cadavers and water displacement, a method that limits the number of subjects drastically and thereby disregards the influence of interindividual variation [2, 15, 20]. Further, some studies provide very little or no demographic information about the subjects, making comparisons even more uncertain [2, 15]. Besides, a substantial portion of anatomical studies on the infraspinatus muscle discusses other size measurements rather than volume; for example, muscle length, muscle mass, and PCSA (physiological cross-sectional area) [20, 27, 32]. With these reservations declared, we consider our reported volume to be fairly in resonance with previous studies, if yet somewhat larger [2, 13, 33]. Possible explanations for the discrepancy could be different subject characteristics not considered in our study; for example, different levels of daily physical activity or targeted shoulder exercise. Also, it has been noted by other researchers that muscles tend to shrink in cadaver studies, which could contribute to diverging results between cadaver studies and MRI studies [13].

Another important finding was that muscle volume correlated with both body weight (*r* = 0.72) and height (*r* = 0.61). In reviewing the literature, only one previous study investigating the correlation between muscle volume and body weight was found. This study reported a weaker correlation than the one we found, although exact values of the correlation coefficients are not presented [13]. One research team has investigated the correlation between body weight and height to the volume of the rotator cuff muscles together as a unit and has found strong correlations among adult males (*r* = 0.63 and *r* = 0.88) [12]. It has also been shown that height correlates with the lateromedial diameter of the infraspinatus muscle [6]. Altogether, the current study's findings broadly support the general picture that there are significant correlations between demographic data and the muscle volume of the infraspinatus. However, larger studies are needed to evaluate demographic factors that are most relevant to consider. Our study indicates that both body weight and height are pertinent, but there are likely more factors not taken into consideration in this study that should be evaluated.

The current study found no correlation between muscle volume and age. One earlier study has indicated that the volume of the infraspinatus muscle increases with age in young males between 10 and 23 years of age [33]. It is reasonable to think that this increase is related to the general body growth occurring during the pre-pubertal and pubertal years. The subjects in our study were of mixed genders and older. Viewing the findings of these two studies together, one might speculate that the infraspinatus growth related to puberty may be over about the age of 20 years and suggest that the infraspinatus volume in young adults has a stronger relationship to body weight and height than to age, as discussed above. Further studies are needed to determine the accuracy of that theory, since other explanations also are possible.

### The angle between the most inferior tendon and the scapular spine

The intention behind measuring the angle between the most inferior tendon and the scapular spine was to indicate the direction of the tendons' course. Interestingly, the measured angle varied substantially among the subjects (5.8°–39.2°) but did not correlate with the number of intramuscular tendons as one could intuitively believe. No correlation between the angle and demographic data was found, which was less surprising.

One could speculate that the measured angle might reflect the width of the tendinous insertion of the infraspinatus muscle at the humeral head. A wide angle may reflect a broad insertion occupying a large portion of the greater tubercle and a narrow angle a slender insertion occupying only a smaller portion of the humeral head. This is intuitive to think when examining pictures such as those shown in Figs. 13 and 14. Recently, Mochizuki et al. [24] conducted a cadaver study indicating that the tendinous insertion of the infraspinatus covers a larger portion of the greater tubercle than what traditionally has been believed. Regarding that, a natural next step would be to measure the width of the tendinous insertion on MRI and thereby investigate whether the results presented by Mochizuki et al. [24], can be replicated. It would also be possible to analyze whether the angle we measured and the insertion width correlate. Further, one could wonder if muscles with different widths of insertions have different functional properties and whether certain variants are related to rotator cuff tendinopathy. We opine that these hypotheses should be objectives for future research.

### Localization of the intramuscular tendons

In 11 examinations, partly superficial intramuscular tendons were identified. Considering how low the prevalence was and how similar the occurrence was in the compared groups, it was not surprising that no correlation to side or gender was found. We advise the reader to note the existence of different positions of the intramuscular tendons as an observation of the yet unclear clinical value of this anatomical variant. However, it could be hypothesized that the position of the intramuscular tendons affect the muscle's force-generating capacity. It is generally accepted that the force-generating capacity is directly proportional to the PCSA [20, 21]. The PCSA can be estimated based on muscle mass, muscle fiber length and pennation angle, i.e., the angle between the muscle fibers and the tendon axis [28]. Since the muscle fibers emanate from the intramuscular tendons [16], it is possible that the position of the intramuscular tendons affect the pennation angle and thereby the PCSA and the muscle's force-generating capacity.

### Strengths and weaknesses

MRI as a technique for studying internal muscle structure might be considered both a great asset and a challenge. A limitation is that the sequences are chosen without consideration to how well the studied variables are captured. This resulted in an unexpected amount of missing data regarding muscle volume in our study, as described in the results section. Another limitation is that no distinct boundary between the infraspinatus and teres minor could be made in the sagittal projections. For this reason, volume calculation was done on the infraspinous fossa rather than on the infraspinatus muscle itself. Therefore, part of the teres minor was likely included in the reported volume. However, the infraspinatus occupies a large portion of the infraspinatus fossa and has a considerably greater muscle volume than the teres minor [15], making our calculation a fair approximation. The same approximation has been made by other researchers [13]. It should be emphasized that this did not affect the reported number of intramuscular tendons since they were counted in the coronal view where the infraspinatus and teres minor were clearly separable.

The distribution of demographics (presented in Table 5) was fairly representative of the corresponding age group of the normal population as reported by Statistics Sweden [5]. However, it should be noted that body weight and height were self-reported variables, which implies a certain amount of incertitude. Our subjects had a slightly higher mean body weight and mean height than the normal population, but this was expected seeing that more men than women were included. However, one may wonder if this skewness in gender distribution among the included subjects reflects that more men than women were referred for an MRI examination at the actual hospital. This would be an unanticipated observation since shoulder complaints generally are more prevalent among women [31]. Another limitation of this study was that no information on handedness was available. The current study found no difference in any of the variables between the right and left sides, but it would have been relevant to investigate if the same result had been obtained if the dominant and non-dominant sides had been compared instead.

The current study could be underpowered to detect some existing correlations and differences between groups. Another remark is that the lateromedial diameter had a slightly skewed distribution, violating the assumptions for ICC calculation.

## Conclusions

The anatomical variations of the infraspinatus muscle are widespread. In the medial part of the muscle belly, the number of intramuscular tendons varied between 3 and 8, while the number of intramuscular tendons laterally varied between 1 and 5. Results of our study may help to understand the internal structure of the infraspinatus muscle and its function in shoulder stabilization.

The most interesting finding was that there was a wide individual variability in the number of intramuscular tendons, muscle volume, and the angle between the most inferior tendon and the scapular spine. This observation raises the question of whether different anatomical variants are associated with different functional properties. Different functional properties might indicate that physiotherapy for patients with rotator cuff tendinopathy should be individually adapted concerning the internal structure of the infraspinatus muscle. Consequently, a natural step in developing more efficient shoulder rehabilitation programs would be to explore whether there is a correlation between anatomical variants and physiotherapy outcomes. Further, it would be interesting to investigate whether different anatomical variants are associated with rotator cuff tendinopathy.

## Data Availability

The datasets generated during and/or analysed during the current study are available from the corresponding author on reasonable request.

## Abbreviations

BMI: Body mass index
FOV: Field of view
ICC: Intraclass correlation coefficient
MRI: Magnetic resonance imaging
PACS: Picture archiving and communication system
PD: Proton density
SD: Standard deviation
SEM: Standard error measurement
SPAIR: Spectral adiabatic inversion recovery
SPSS: Statistical package for the social sciences
TE: Echo time
TR: Repetition time
TSE: Turbo spin echo
